# The Effect of Metformin on Diastolic Function in Patients Presenting with ST-Elevation Myocardial Infarction

**DOI:** 10.1371/journal.pone.0168340

**Published:** 2016-12-15

**Authors:** Lawien Al Ali, Minke T. Hartman, Chris P. H. Lexis, Yoran M. Hummel, Erik Lipsic, Joost P. van Melle, Dirk J. van Veldhuisen, Adriaan A. Voors, Iwan C. C. van der Horst, Pim van der Harst

**Affiliations:** 1 Department of Cardiology, University of Groningen, University Medical Center Groningen, Groningen, the Netherlands; 2 Department of Critical Care, University of Groningen, University Medical Center Groningen, Groningen, the Netherlands; Universita degli Studi di Perugia, ITALY

## Abstract

**Introduction:**

Diastolic dysfunction is an important predictor of poor outcome after myocardial infarction. Metformin treatment improved diastolic function in animal models and patients with diabetes. Whether metformin improves diastolic function in patients presenting with ST-segment elevation myocardial infarction (STEMI) is unknown.

**Methods:**

The GIPS-III trial randomized STEMI patients, without known diabetes, to metformin or placebo initiated directly after PCI. The previously reported primary endpoint was left ventricular ejection fraction at 4 months, which was unaffected by metformin treatment. This is a predefined substudy to determine an effect of metformin on diastolic function. For this substudy trans-thoracic echocardiography was performed during hospitalization and after 4 months. Diastolic dysfunction was defined as having the combination of a functional alteration (i.e. decreased tissue velocity: mean of septal e’ and lateral e’) and a structural alteration (i.e. increased left atrial volume index (LAVI)). In addition, left ventricular mass index and transmitral flow velocity (E) to mean e' ratio (E/e’) were measured to determine an effect of metformin on individual echocardiographic markers of diastolic function.

**Results:**

In 237 (63%) patients included in the GIPS-III trial diastolic function was measured during hospitalization as well as at 4 months. Diastolic dysfunction was present in 11 (9%) of patients on metformin and 11 (9%) patients on placebo treatment (*P* = 0.98) during hospitalization. After 4 months 22 (19%) of patients with metformin and 18 (15%) patients with placebo (*P* = 0.47) had diastolic dysfunction. In addition, metformin did not improve any of the individual echocardiographic markers of diastolic function.

**Conclusions:**

In contrast to experimental and observational data, our randomized placebo controlled trial did not suggest a beneficial effect of short-term metformin treatment on diastolic function in STEMI patients.

## Introduction

Diastolic dysfunction is common after acute myocardial infarction and is estimated to affecting up to 40% of patients [1–3]. It is also an important and independent predictor of adverse outcome, irrespective of and even in the absence of systolic dysfunction [3–11]. The presence of (pre)diabetes, the extent of myocardial injury, delayed and unsuccessful reperfusion, a history of hypertension and female sex have been associated with diastolic dysfunction after myocardial infarction [11–17]. Acute myocardial injury has been associated with direct regional diastolic dysfunction and the subsequent infarct healing and remodeling can affect global diastolic function [11,18]. There is no therapy available to treat or prevent the occurrence of diastolic dysfunction after myocardial infarction [19–21].

Metformin is the most widely used oral antihyperglycemic agent for the treatment of type 2 diabetes [22]. In diabetics, metformin has been associated with improved outcome independently of glycemic control, and there are indications it may have direct cardioprotective effects [22–27]. The use of metformin has been associated with improved diastolic function in patients with diabetes undergoing coronary angiography [28]. In dogs with pacing-induced heart failure, metformin significantly reduced capillary wedge pressure and left ventricular end-diastolic pressure compared to control [29]. Metformin also lowered left ventricular end-diastolic pressure in a non-diabetic rat model [30]. These studies led to the hypothesis that metformin treatment might reduce the risk of diastolic dysfunction development in patients presenting with myocardial infarction.

In the Glycometabolic Intervention as adjunct to Primary Coronary Intervention in ST-Elevation Myocardial Infarction (GIPS-III) study we investigated the effect of metformin treatment in non-diabetic patients. We previously reported the primary endpoint, left ventricular ejection fraction at 4 months, which was unaffected by metformin treatment [31]. In the present pre-specified sub-study of GIPS-III, we analyzed the effect of metformin treatment on diastolic function in non-diabetic patients undergoing successful primary percutaneous coronary intervention (PCI) for ST-segment elevation myocardial infarction (STEMI).

## Methods

The current echocardiographic sub-study is a predefined ancillary study of the GIPS-III trial. The GIPS-III trial has been registered as a clinical trial with identifier: NCT01217307. The GIPS-III trial was a single center, randomized, double blind, placebo-controlled study. The study design, baseline characteristics and primary outcomes have previously been reported [31,32]. In short, patients presenting with STEMI to the University Medical Center Groningen between January 1, 2011, and May 26, 2013 who underwent successful PCI with implantation of at least 1 stent with a diameter of at least 3 mm were eligible for inclusion in the trial. Patients also needed to be able to undergo magnetic resonance imaging to assess the primary endpoint (LVEF) and had to be 18 years of age or older. Major exclusion criteria included known diabetes, previous myocardial infarction, and severe renal dysfunction. Patients were randomized to either metformin 500mg twice daily or a visually matching placebo twice daily, starting immediately after PCI. Verbal informed consent was obtained from the patients during the PCI and written consent followed after admission to the Coronary Care Unit (CCU). After four months of treatment patients underwent cardiac magnetic resonance imaging (MRI) to assess the primary endpoint, LVEF. The study protocol was approved by the local ethics committee (Groningen, the Netherlands), and was in accordance with the Declaration of Helsinki [33] and Dutch laws.

### Echocardiography

A trans-thoracic echocardiogram in left decubital position was performed by experienced operators using a Vivid 7 echo system (General Electric, Horton, Norway) during hospitalization for the index event and at four months follow-up. All echocardiographic data were digitally stored in DICOM format and off-line analysis was performed by experienced sonographers on an Echopac BT 10 (General Electric, Horton, Norway) at an independent core lab (Groningen Imaging Core Laboratory, Groningen, the Netherlands). Observers performing the analyses were blinded to treatment allocation and clinical information. Echocardiographic evaluation was performed as recommended by the guidelines in effect [34–39]. When appropriate measures were indexed for body surface area (BSA) according to the formula of Du Bois and Du Bois [40]. The following structural measurements were assessed: Left ventricular (LV) interventricular septal and posterior wall thickness (IVS and LVPW), left ventricular end-diastolic (LVEDD) and end-systolic diameter (LVESD). In addition, Simpson’s biplane volumetric measurements including LV end-diastolic volume (LVEDV) and end-systolic volume (LVESV). LVEF was calculated as LVEF = (LVEDV-LVESV)/LVEDV x 100%. Left atrial volume (LAV) was measured with the area length method. The LV mass was estimated from linear dimensions as suggested by Devereux and colleagues [37]. LV diastolic parameters included Doppler measurement of the passive early filling (E), active atrial filling (A), isovolumetric relaxation time (IVRT) and E wave deceleration time (DT). Using spectral tissue Doppler, early diastolic tissue velocities (e’) from both the septal and lateral wall were assessed [35,36,41–43]. Mean e’ was calculated as (e’ septal + e’ lateral)/2. Reported values represent the mean of three heart beats in end-expiration.

Individual parameters of diastolic function were interpreted as recommended by the latest guidelines from the European Society of Cardiology and the American Society of Echocardiography: Mean e’ was deemed abnormal if ≤9cm/s; E/e’ was calculated as E/mean e’ and was deemed abnormal if ≥13; indexed left atrial volume (LAVI) was deemed abnormal if ≥34 ml/m^2^; left ventricular mass index (LVMI) was deemed abnormal if ≥95 gram/m^2^ in women and ≥115 gram/m^2^ in men, mean e’ was deemed abnormal if ≤9cm/s, and E/e’ was deemed abnormal if ≥13 [39,44].

The definition of diastolic dysfunction was based on the recommendations made by Nagueh and colleagues as well as recommendations made by the European Society of Cardiology [34,39]. The presence of diastolic dysfunction was defined as having a LAVI larger than 34ml/m^2^ in combination with a mean e’ < 9cm/s.

### Statistical analysis

The primary analysis was the presence of diastolic dysfunction in patients in the metformin group compared to the placebo group. The individual parameters of diastolic function in both groups were analyzed as a secondary analysis.

Continuous variables are presented as mean ± standard deviation (SD) for values that approximate a normally distribution and as median with their interquartile range (IQR) if values do not approximate a normal distribution. For continuous variables differences between groups were tested using 2-tailed t test for normally distributed data and Wilcoxon rank-sum for non-normally distributed data. For binary variables differences were assessed using the Pearson’s chi-squared test and an ordered logistic regression for ordinal variables. All reported P values are 2-sided, and a P-value of <0.05 was considered to indicate significant difference between groups. All analyses were performed using Stata version 14.1 (StataCorp).

## Results

**Fig 1:**
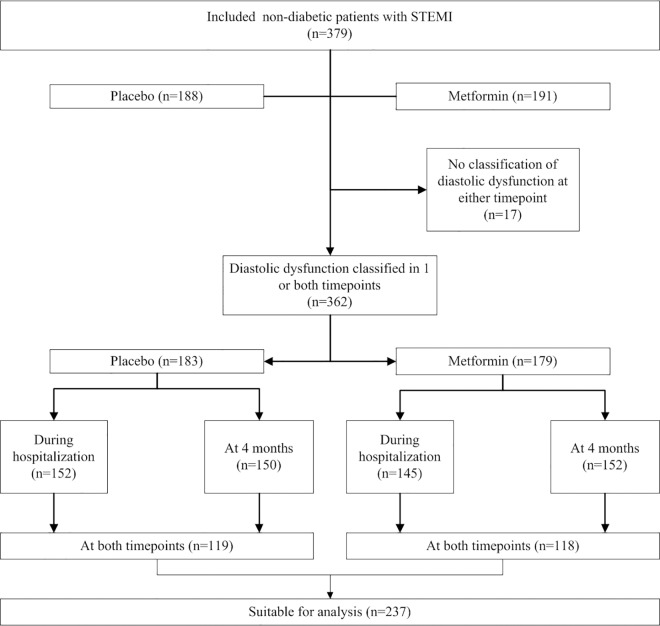
Flowchart of echocardiographic assessment during hospitalization and at 4 months according to randomization. STEMI denotes ST-segment elevation myocardial infarction.

Three hundred and seventy-nine patients participated in the GIPS-III trial and at 4 months after infarction, all patients were alive and none were lost to follow-up. Of these patients 329 (87%) underwent echocardiography both during hospitalization and at 4 months; 43 (11%) patients only underwent echocardiography during hospitalization and 5 (1.3%) patients only at 4 months. Of the 706 echocardiographic procedures that were performed, the presence of diastolic dysfunction could be reliably classified in 599 (85%) procedures. The presence of diastolic dysfunction could be classified only during hospitalization in 60 (16%) patients, and only at 4 months in 65 (17%) patients. The presence of diastolic dysfunction could be classified at both time points in 237 (64%) patients (Fig 1).

**Fig 2:**
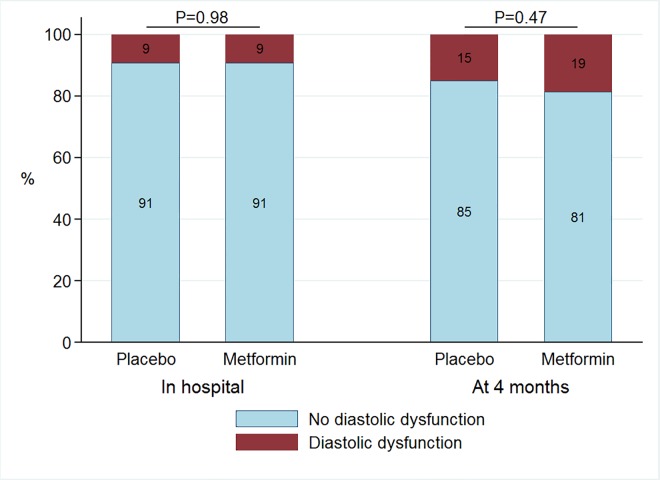
Barchart of classification of diastolic function during hospitalization and at 4 months according to randomization.

**Fig 3:**
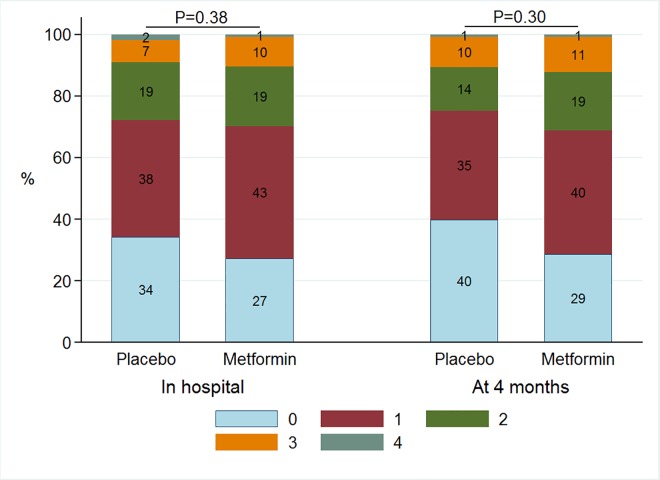
Barchart of prevalence of abnormal individual parameters of diastolic function during hospitalization and at 4 months according to randomization.

The baseline characteristics for these 237 patients in whom the presence of diastolic dysfunction could be classified at both time points are presented in Table 1. The two treatment groups were well balanced for baseline characteristics. Also, we did not observe a substantial baseline differences between subjects with available echocardiographic measurements of diastolic dysfunction compared to those without (S1 Table). Diastolic dysfunction was present in 22 (9%) of the 237 patients for whom the presence of diastolic dysfunction could be classified at both time points. During hospitalization 11 (9%) patients in the metformin group had diastolic dysfunction compared with 11 (9%) patients in the placebo group (*P* = 0.98) (Fig 2). During hospitalization, measurements of cardiac structure and function for patients treated with metformin (n = 118) or placebo (n = 119) were similar (Table 2). During hospitalization 83 (73%) patients in the metformin group had at least one abnormal diastolic parameter compared with 73 (66%) patients in the placebo group (P = 0.25). Treatment with metformin versus administering a placebo did not have effect on the distribution of the amount of abnormal parameters of diastolic function during hospitalization (P = 0.38) (Fig 3).

**Table 1:** Baseline characteristics of the population that underwent echocardiography in hospital and at 4 months by treatment allocation.

		No.(%)		
Characteristic	Total (n = 237)	Placebo (n = 119)	Metformin (n = 118)	*P*-value
Age, years	58.1 ± 11.0	58.2 ± 10.7	57.9 ± 11.4	0.82
Women	53 (22.4%)	30 (25.2%)	23 (19.5%)	0.29
Body weight, kg	84.4 ± 14.0	84.3 ± 13.7	84.5 ± 14.3	0.88
Body-mass Index, kg/m^2^	26.8 ± 3.5	26.9 ± 3.3	26.7 ± 3.7	0.61
Race/ethnicity				
White	230 (97.0%)	116 (97.5%)	114 (96.6%)	0.69
Asian	6 (2.5%)	3 (2.5%)	3 (2.5%)	0.99
Black	1 (0.4%)	0 (0.0%)	1 (0.8%)	0.50
Cardiovascular related history				
Hypertension	66 (27.8%)	31 (26.1%)	35 (29.7%)	0.54
Dyslipidemia	154 (65.0%)	83 (69.7%)	71 (60.2%)	0.12
Current smoking	124 (52.3%)	59 (49.6%)	65 (55.1%)	0.40
Stroke	1 (0.4%)	1 (0.8%)	0 (0.0%)	1.00
Previous PCI	4 (1.7%)	3 (2.5%)	1 (0.8%)	0.32
Blood pressure, mmHg				
Systolic	134.7 ± 23.2	134.7 ± 23.7	134.6 ± 22.8	0.98
Diastolic	85.1 ± 14.4	85.1 ± 14.8	85.1 ± 14.0	0.98
Heart rate, beats/min	75.3 ± 16.0	76.4 ± 17.4	74.2 ± 14.5	0.29
Infarct-related factors				
Ischemia time, min	152 (107, 245)	144 (105, 232)	169 (109, 264)	0.23
Single vessel disease	166 (70.0%)	87 (73.1%)	79 (66.9%)	0.30
Anterior infarction	95 (40.1%)	46 (38.7%)	49 (41.5%)	0.65
Intervention-related assesments				
TIMI flow grade pre PCI ≤ 1	154 (65.0%)	84 (70.6%)	70 (59.3%)	0.07
TIMI flow grade post PCI < 3	14 (5.9%)	6 (5.0%)	8 (6.8%)	0.57
Myocardial blush grade ≤ 1	20 (8.5%)	8 (6.7%)	12 (10.3%)	0.32
Laboratory values at admission				
Glucose, mmol/l	8.2 (7, 9.6)	8.4 (7, 10.1)	8.1 (6.8, 9.4)	0.31
HbA_1c_, %	5.8 (5.6, 6)	5.8 (5.6, 6)	5.75 (5.6, 6)	0.73
Hemoglobin, mmol/l	9 (8.4, 9.4)	8.9 (8.3, 9.5)	9 (8.5, 9.3)	0.54
Creatinine, μmol/l	72 (62, 81)	73 (64, 80)	71 (60, 82)	0.82
eGFR, ml/min/1.73m^2^	96 (87, 103)	95 (88, 101)	97 (86, 105)	0.22
NT-proBNP, ng/L	78 (40, 177)	68 (37, 177)	79.5 (42, 179)	0.50
CK, U/L	129 (85, 192)	123 (82, 179)	132 (89, 218)	0.20
Myocardial band of CK, U/L	16 (13, 23)	15 (12, 22)	16 (13, 27)	0.16
Troponine T, ng/L	43 (21, 115)	39.5 (22, 87)	49 (20, 136)	0.45
Total cholesterol, mmol/l	5.4 (4.8, 6)	5.45 (4.9, 6)	5.35 (4.7, 6.1)	0.71
LDL cholesterol, mmol/l	3.8 (3.25, 4.4)	3.8 (3.3, 4.4)	3.7 (3.2, 4.6)	0.96
HDL cholesterol, mmol/l	1.1 (0.9, 1.4)	1.1 (0.9, 1.4)	1.1 (0.9, 1.3)	0.67

SD, standard deviation; PCI, percutaneous coronary intervention; IQR, interquartile range; TIMI, Thrombolysis in Myocardial Infarction; HbA1c, glycated hemoglobin; NT-proBNP, N-terminal pro brain natriuretic peptide; eGFR, estimated glomerular filtration rate; CK, creatine kinase; LDL, low density lipoprotein; HDL high density lipoprotein.

**Table 2:** Echocardiographic measurements during hospitalization and at 4 months.

	During hospitalization			At 4 months			Change between both visits		
Variable	Placebo (n = 119)	Metformin (n = 118)	*P*-value	Placebo (n = 119)	Metformin (n = 118)	*P*-value	Placebo (n = 119)	Metformin (n = 118)	*P*-value
Time until echo, days	2 (1, 3)	2 (1, 3)	0.29	124(119, 128)	123 (116, 128)	0.41	121 (116, 127)	120 (11, 126)	0.13
LVEDV, ml	93.2 (79.2, 121.3)	102.5 (83.1, 122.3)	0.21	102.7 (87.1, 122.9)	102.8 (87.6, 129.8)	0.63	5.5 (-3.3, 19.0)	4.6 (-10.6, 22.0)	0.42
LVESV, ml	47.0 (35.4, 62.6)	48.2 (37.0, 62.2)	0.49	41.9 (35.3, 54.1)	45.9 (36.7, 63.9)	0.08	-1.8 (-8.3, 5.4)	-0.3 (-6.8, 8.7)	0.36
LVEF, %	52 (46, 57)	52 (45, 59)	1.00	58 (53, 63)	55 (48, 60)	<0.01	3.7 (0.3, 10.6)	3.2 (-2.9, 7.6)	0.04
LV mass, gram	171.1 (144.2, 200.1)	181.5 (151.6, 214.7)	0.25	173.0 (149.6, 199.1)	180.5 (148.8, 207.8)	0.38	0.2 (-23.4, 18.7)	-0.9 (-31.1, 13.7)	0.46
E, cm/s	0.6 (0.5, 0.8)	0.7 (0.6, 0.8)	0.05	0.6 (0.6, 0.8)	0.7 (0.6, 0.8)	0.60	0.04 (-0.09, 0.16)	-0.01 (-0.10, 0.12)	0.23
A, cm/s	0.6 (0.5, 0.7)	0.6 (0.5, 0.7)	0.77	0.7 (0.6, 0.8)	0.7 (0.5, 0.8)	0.82	0.04 (-0.07, 0.11)	0.02 (-0.08, 0.11)	0.69
E/A ratio	1.0 (0.8, 1.3)	1.0 (0.9, 1.3)	0.27	1.0 (0.8, 1.2)	1.0 (0.8, 1.2)	0.43	-0.01 (-0.18, 0.19)	-0.03 (-0.18, 0.17)	0.45
DT, ms	184.9 (148.1, 215.1)	173.3 (147.1, 217.5)	0.48	210.4 (178.0, 242.3)	199.3 (164.0, 245.3)	0.12	32.8 (-1.6, 69.3)	29.7 (-6.4, 56.2)	0.35
IVRT, cm/s	96.9 (86.5, 103.8)	99.2 (87.7, 117.6)	0.16	99.8 (90.0, 114.3)	96.9 (83.0, 110.7)	0.14	1.2 (-10.4, 18.5)	-4.6 (-18.4, 13.8)	0.19
Septal e`, cm/s	7.7 (6.3, 9.1)	7.9 (6.2, 9.1)	0.94	8.1 (6, 9.5)	7.9 (6.9, 9.4)	0.67	0.1 (-1.0, 1.2)	0.4 (-1.1, 1.7)	0.45
Lateral e`, cm/s	9.8 (7.7, 12)	9.3 (6.9, 12)	0.28	10 (8.5, 12)	10 (7.8, 11)	0.07	0.3 (-1.2, 2.5)	0.0 (-1.2, 1.6)	0.31
Mean e`, cm/s	8.9 (7.1, 10)	8.4 (7, 10)	0.50	9.2 (7.7, 11)	8.9 (7.8, 10)	0.32	0.3 (-0.8, 1.3)	0.2 (-0.6, 1.5)	0.88
E/e`ratio	7.1 (5.9, 9.7)	7.8 (6.7, 9.6)	0.08	7.5 (6.2, 8.7)	7.6 (6.4, 9)	0.51	0.2 (-1.3, 1.9)	-0.1 (-1.3, 1.0)	0.31
LAVI, ml/m^2^	26 (22, 32)	27 (23, 32)	0.47	28 (24, 34)	28 (25, 35)	0.98	1.6 (-2.7, 7.3)	1.2 (-2.7, 6.6)	0.66
LVMI, gram/m^2^	85 (73, 101)	89 (75, 105)	0.22	85.7 (73.6, 97.0)	88.4 (75.7, 100.9)	0.26	0.6 (-13.5, 9.1)	-2.7 (-16.0, 6.9)	0.41

LVEDV, left ventricular end-diastolic volume; LVESV, left ventricular end-systolic volume; LVEF, left ventricular ejection fraction; LV mass, left ventricular mass; E, passive early filling of the left ventricle; A, active atrial filling of the left ventricle; DT, E wave deceleration time; E-wave deceleration time; IVRT isovolumetric relaxation time; Septal e`, early diastolic tissue velocity from septal wall; Lateral e`, early diastolic tissue velocity from lateral wall; LAVI, left atrial volume indexed for body mass; LVMI, left ventricular mass indexed for body mass.

At 4 months after myocardial infarction, diastolic dysfunction was present in 40 (17%) of the 237 patients for whom the presence of diastolic dysfunction could be classified at both time points; 118 patients randomized to metformin and 119 to placebo. In the metformin group 22 (19%) patients had diastolic dysfunction compared to 18 (15%) patients in the placebo group (*P* = 0.47) (Fig 2). At 4 months, diastolic function parameters were similar between groups. Table 2 displays the changes of all individual echocardiographic parameters between baseline and 4 months. Except LVEF, we found no other statistically significant changes in any of the echocardiographic parameters. At 4 months 82 (71%) patients in the metformin group had at least one abnormal diastolic parameter compared with 68 (60%) patients in the placebo group (P = 0.08). Treatment with metformin versus administering a placebo did not have effect on the distribution of the amount of abnormal parameters of diastolic function at 4 months (P = 0.30) (Fig 3).

Change in the classification of diastolic dysfunction was present in 34 (14%) out of 237 patients in whom diastolic dysfunction could be classified at both time points, with diastolic dysfunction developing in 26 (11%) patients and diastolic dysfunction reverting to normal in 8 (8%) patients. In the metformin group 14 (12%) out of 118 patients developed diastolic dysfunction between hospitalization and follow-up and 3 (3%) patient reverted from dysfunction back to normal function compared with 12 (10%) out of 119 patients in the placebo group that developed dysfunction and 5 (4%) patients who reverted from dysfunction back to normal (*P* = 0.54). Also none of the individual measurements related to diastolic function were different between the 175 patients that received 4 months of placebo treatment versus the 173 patients that were treated with metformin.

## Discussion

In this predefined sub study of a prospective, randomized, placebo controlled trial, we found that metformin did not improve diastolic function as compared to placebo in patients presenting with STEMI. These findings are in contrast with earlier observational and experimental studies suggesting beneficial effects of metformin treatment on diastolic function. In a rat model of ischemia-reperfusion injury, metformin treatment resulted in lower left ventricular end-diastolic pressure, independent from glycemic control [30]. Also in dogs with pacing induced heart failure, metformin treatment improved diastolic function as indicated by a reduced left ventricular end-diastolic pressure [29]. In patients with diabetes, improvements of glycemic control, including the use of metformin, was associated with improvements of diastolic function as measured by septal e’ [45]. Also in the setting of stable coronary artery disease, metformin use in diabetic patients was associated with improved left ventricular diastolic function as measured by mean e’ and IVRT [28].

The frequency of diastolic dysfunction during hospitalization and follow-up in our study was substantial but less than reported by others. Other studies have reported substantially higher numbers of diastolic dysfunction after myocardial infarction [4,7,9,46–48]. Differences in the population might explain some of the prevalence of diastolic dysfunction. In our study, patients with known diabetes were excluded and diabetes is associated with diastolic dysfunction [38]. Also the prevalence of insulin resistance and the metabolic syndrome, which are closely related to diastolic dysfunction, was low in the GIPS-III trial. Especially in patient with the metabolic syndrome, increasing insulin sensitivity by metformin might be associated with improvements of diastolic function. MET-DIME is an ongoing prospective trial in which metformin treatment will be tested in patients without diabetic but with the metabolic syndrome [49]. The primary endpoint of MET-DIME is the change in mean of early diastolic mitral annular velocity.

Another important difference which might explain differences in the reported prevalence of diastolic function after myocardial infarction can be attributed to the definitions used to designate diastolic dysfunction. No single echocardiographic parameter can be effectively used to make a diagnosis of LV diastolic dysfunction [19]. However, several previous studies defined diastolic function as a single echocardiographic parameter.

In contrast, our definition was based on the presence of both increased LAVI as well as decreased mitral annular tissue Doppler velocity. This definition combines a finding of structural abnormality related to diastolic function with a finding of functional abnormality related to diastolic function as is recommended by Nagueh and colleagues as well as the most recent guidelines of the European Society of Cardiology [34,39].

The prevalence of abnormality in single echocardiographic parameters for diastolic function was substantially higher in our cohort. The sub-study from the Cardiac Arrhythmias and Risk Stratification after Acute Myocardial Infarction (CARISMA) study also graded diastolic dysfunction based on a combined assessment of E′, DT, E/A ratio, and IVRT. They excluded LAVI in their definition and reported diastolic dysfunction in 39% of patients, six weeks after MI. However, a major difference with our study is that the CARISMA study only included patients with an LVEF <40%[4]. Reduced LVEF is also associated with the presence of diastolic dysfunction [3]. A meta-analysis of over 3000 patients after acute myocardial infarction with a mean LVEF of 46% observed diastolic dysfunction in approximately 20% of patients. In the subgroup of patients with LVEF>53% the prevalence of diastolic dysfunction was only 9% [3]. Considering the mean LVEF of our trial (51%) these numbers are comparable.

In the present subgroup, a 3% lower LVEF was observed in the metformin compared with placebo patients. We previously reported that metformin has no effect on the outcome of LVEF [31]. It is important to note that the current reported LVEF is based on the echocardiographic measurement of a subgroup of the GIPS3 trial while the previously reported LVEF was based on MRI and was defined as the primary endpoint. MRI is considered superior to echocardiography and is currently considered the reference standard for determination of the LVEF [50].

The major strength of this study is that the GIPS-III is a prospective, randomized, placebo controlled trial in which we a priory defined diastolic function as a secondary endpoint. However, some limitations exist. We cannot exclude the metformin dosage was too low or the duration of treatment too short to have measurable effect on diastolic function. Our trial was not powered for diastolic function parameters and although we studied diastolic function as a continuous variable, the distribution of diastolic function might be too narrow in our cohort. We cannot exclude that metformin might be effective in patients with diabetes. Also the patient population was at low risk for developing diastolic dysfunction leading to a relatively low number of cases and these post-hoc analyses might be underpowered to detect small differences. Furthermore, since evaluation of ischemia was not part of the study protocol we cannot exclude that a concomitant effect of myocardial ischemia and/or percutaneous revascularization may have influenced our analyses. Also we cannot exclude that pre-existing diastolic dysfunction was present in some patients and may have influenced our analyses. Roughly one third of the original cohort was excluded because echocardiography was not performed or because echocardiography did not allow for diastolic measurements, although we did not find evidence, we cannot exclude that a certain patient selection based on presence of echocardiographic measurements has influenced our results.

In conclusion, the prevalence of diastolic dysfunction after first STEMI in non-diabetic patients was approximately 16%. In contrast to previous experimental and observational data, our placebo controlled, randomized trial data did not suggest a beneficial effect of 4 months of metformin treatment on diastolic function parameters when initiated after PCI for STEMI.

## Supporting Information

S1 Table Baseline characteristics of study population by presence of measurements of diastolic dysfunction in hospital. (DOCX)
