# Supplementary material for: The Effect of Metformin on Diastolic Function in Patients Presenting with ST-Elevation Myocardial Infarction
Source: PLoS One. 2016 Dec 15;11(12):e0168340. doi: 10.1371/journal.pone.0168340 (PMC5158040; doi:10.1371/journal.pone.0168340)
Supplement: S1 Table — (DOCX) [file pone.0168340.s001.docx]

**Supporting Information**

**S1 Table. Baseline characteristics of study population by presence of measurements of diastolic dysfunction in hospital.**

|  | **No.(%)** | |  |
| --- | --- | --- | --- |
| **Characteristic** | **No measurements (n=82)** | **Measurements present (n=297)** | ***P*-value** |
| Randomized to metformin | 46 (56.1%) | 145 (48.8%) | 0.24 |
| Age, years | 59.9 ± 12.1 | 58.5 ± 11.5 | 0.35 |
| Women | 20 (24.4%) | 75 (25.3%) | 0.87 |
| Body weight, kg | 85.5 ± 15.4 | 83.8 ± 14.0 | 0.35 |
| Body-mass Index, kg/m^2^ | 27.6 ± 4.6 | 26.8 ± 3.6 | 0.09 |
| Race/ethnicity |  |  |  |
| White | 77 (93.9%) | 288 (97.0%) | 0.19 |
| Asian | 3 (3.7%) | 7 (2.4%) | 0.52 |
| Black | 2 (2.4%) | 2 (0.7%) | 0.21 |
| Cardiovascular related history |  |  |  |
| Hypertension | 28 (34.1%) | 84 (28.3%) | 0.30 |
| Dyslipidemia | 48 (58.5%) | 191 (64.3%) | 0.34 |
| Current smoking | 50 (61.0%) | 159 (53.5%) | 0.23 |
| Stroke | 1 (1.2%) | 2 (0.7%) | 0.52 |
| Previous PCI | 0 (0.0%) | 4 (1.3%) | 0.29 |
| Blood pressure, mmHg |  |  |  |
| Systolic | 134.9 ± 22.7 | 134.2 ± 23.6 | 0.83 |
| Diastolic | 84.5 ± 13.4 | 84.3 ± 14.9 | 0.93 |
| Heart rate, beats/min | 76.9 ± 17.4 | 75.4 ± 16.0 | 0.44 |
| Infarct-related factors |  |  |  |
| Ischemia time, min | 156.5 (109, 249) | 161 (109, 251) | 0.81 |
| Single vessel disease ­ | 53 (64.6%) | 205 (69.0%) | 0.45 |
| Anterior infarction | 33 (40.2%) | 113 (38.0%) | 0.72 |
| Intervention-related assesments |  |  |  |
| TIMI flow grade pre PCI ≤ 1 | 49 (59.8%) | 186 (62.6%) | 0.64 |
| TIMI flow grade post PCI < 3 | 12 (14.6%) | 22 (7.4%) | 0.04 |
| Myocardial blush grade ≤ 1 | 12 (14.6%) | 27 (9.2%) | 0.15 |
| Laboratory values at admission |  |  |  |
| Glucose, mmol/l | 8.1 (7.1, 9.6) | 8.2 (7, 9.55) | 0.90 |
| HbA_1c_, % | 5.9 (5.6, 6.1) | 5.8 (5.6, 6) | 0.04 |
| Hemoglobin, mmol/l | 9.1 (8.6, 9.4) | 8.9 (8.4, 9.4) | 0.45 |
| Creatinine, µmol/l | 73 (63, 84) | 72 (62, 81) | 0.56 |
| eGFR, ml/min/1.73m^2^ | 95 (79, 103) | 96 (86, 103) | 0.64 |
| NT-proBNP, ng/L | 84.5 (39, 196) | 81 (40, 200) | 0.60 |
| CK, U/L | 141 (86, 283) | 128 (83, 189) | 0.14 |
| Myocardial band of CK, U/L | 16.5 (12, 29) | 16 (13, 24) | 0.65 |
| Troponine T, ng/L | 58.5 (29, 163) | 49 (23, 128) | 0.16 |
| Total cholesterol, mmol/l | 5.3 (4.6, 5.7) | 5.4 (4.8, 6.1) | 0.24 |
| LDL cholesterol, mmol/l | 3.7 (3.2, 4.3) | 3.8 (3.2, 4.5) | 0.27 |
| HDL cholesterol, mmol/l | 1.1 (1.0, 1.3) | 1.1 (0.9, 1.3) | 0.67 |

SD, standard deviation; PCI, percutaneous coronary intervention; IQR, interquartile range; TIMI, Thrombolysis in Myocardial Infarction; HbA1c, glycated hemoglobin; NT-proBNP, N-terminal pro brain natriuretic peptide; eGFR, estimated glomerular filtration rate; CK, creatine kinase; LDL, low density lipoprotein; HDL high density lipoprotein.
